# Initial validation of the Italian version of the Volition in Exercise Questionnaire (VEQ-I)

**DOI:** 10.1371/journal.pone.0249667

**Published:** 2021-04-09

**Authors:** Maria Chiara Gallotta, Valerio Bonavolontà, Laura Guidetti, Carlo Baldari, Lorenzo Innocenti, Ludovica Cardinali, Lavinia Falcioni, Selenia di Fronso, Gian Pietro Emerenziani, Giovanna Zimatore, Maurizio Bertollo

**Affiliations:** 1 Department of Physiology and Pharmacology “Vittorio Erspamer”, Sapienza University of Rome, Rome, Italy; 2 Department of Basic Medical Sciences, Neuroscience and Sense Organs, University of Bari “Aldo Moro”, Bari, Italy; 3 Università degli Studi Niccolò Cusano, Rome, Italy; 4 eCampus University, Novedrate, Italy; 5 Department of Movement, Human and Health Sciences, University of Rome “Foro Italico”, Rome, Italy; 6 Department of Medicine and Aging Sciences, University G. d’Annunzio of Chieti-Pescara, Chieti, Italy; 7 Department of Experimental and Clinical Medicine, “Magna Græcia” University of Catanzaro, Catanzaro, Italy; Università degli Studi di Perugia, ITALY

## Abstract

The purpose of this study was to validate the Volition in Exercise Questionnaire in Italian language (VEQ-I). The translation and cultural adaptation of the VEQ-I was conducted using the forward-backward translation method. VEQ-I eighteen items correspond to the six-factors structure of the original version. The construct validity was verified by the confirmatory factor analysis (CFA) (CFI = 0.960; TLI = 0.943; RMSEA = 0.039; and SRMR = 0.040). The eighteen items were well distributed in six subscales and the six-factors structure of the questionnaire was supported. Internal Consistency value of the questionnaire was investigated for each subscale of the VEQ-I. Cronbach’s alpha and Omega values of the *Reasons*, *Postponing Training*, *Unrelated Thoughts*, *Self-Confidence*, *Approval from Others* and *Coping with Failure* subscales were 0.76 (α) and 0.76 (ω), 0.76 (α) and 0.76 (ω), 0.87 (α) and 0.88 (ω), 0.85 (α) and 0.85 (ω), 0.70 (α) and 0.72 (ω) and 0.74 (α) and 0.74 (ω), respectively. They were acceptable in all the six subscales. The concurrent validity was assessed using the correlation among the subscales of VEQ-I measures and those contained in two questionnaires: Psychobiosocial States in Physical Education (PBS-SPE) and Exercise Motivations Inventory (EMI-2).

## Introduction

There is a growing body of literature suggesting that regular practice of physical activity can improve individuals’ health, quality of life, physical and emotional well-being [1,2], reducing the incidence and the implications of many diseases and disabilities [3–6]. Despite this scientific evidence, globally, one out of four adults do not meet the global recommended levels of physical activity [7]. This alarming world scenario appears also in Italy. In fact, nearly 40% of the Italian adult population is inactive [8] and physical inactivity can be considered one of the most serious public health issues of contemporary times [9].

Therefore, adherence to physical activity seems to have a key role to gain or to maintain a healthy lifestyle in the general population. Many studies have investigated psychological and motivational factors that influenced the participation of individuals in regular physical activity.

During the past 40 years much progress has been achieved in this field, moving from those studies on the psychological predictors for participation in physical exercise [10–12] to the factors and strategies that can drive behaviour [13], focusing on different motivational theories such as the i) self-determination theory [14], ii) the theory of planned behaviour [15] and iii) the social cognitive theory [16].

The self-determination theory is an empirically derived theory of human motivation and personality in social contexts that describes the motivation in terms of being autonomous and controlled. Work leading to the theory began with experiments examining the effects of extrinsic rewards on intrinsic motivation [14].

The concept of the theory of planned behaviour was proposed to improve the predictive power of the theory of reasoned action by including perceived behavioural control. This theory associates one’s beliefs and behaviour [15].

The social cognitive theory considers the unique way in which individuals acquire and maintain behaviour, while also considering the social environment where the individuals perform the behaviour. This theory considers how past experiences influence reinforcements, expectations whether a person will engage in a specific behaviour and the reasons why a person engages in that behaviour [16]. Therefore, person’s motivation seems to be the stimulus for behaviour change that needs an applied model of intervention. For instance, the stage models highlight the psychological and behavioural processes that an individual has in invoking behaviour change [17], all of which are highly nuanced and include multiple theoretical underpinnings such as the motivational framework previously described. Motivation is an important factor for adopting and maintaining physical exercise and also intention is a central influencing factor for physical exercise behavior [18]. Therefore, besides motivational processes that lead to the intention of a behavioral change, volitional processes are deemed necessary to transform intention into concrete action, leading to the actual behavior [19]. One of the most adopted models of behaviour change, which might be applied for motivating people towards an active lifestyle and the adoption of physical activity habit is the trans-theoretical model [20]. The model has been successfully utilised to describe the different phases that people adopt in the acquisition and maintenance of physical activity behaviours. The trans-theoretical model provides a framework for categorising a person’s readiness to change their behaviour and includes five stages. In this theoretical framework, a greater extent by autonomous or volitional motives, rather than by controlled or pressured motives is expected, and motivation per se is not sufficient in moving from one stage (or phase) to the other.

Similarly, the Heckhausen’s Rubicon model of action phases (1991) suggest that motivation only imply part of the cognitive processes of an acting individual, including thinking about the consequences of this action. According to the Rubicon model, every action includes such a point of no return at which the individual moves from goal setting to goal striving. The cognitive processes in this framework include both motivation and volition [21,22]. Volition goes beyond comprehension on the laudable of goals and explains how actions are applied and finalized [23]. The concept of volition refers to an individual’s self-regulatory mental process responsible for originating and preserving a desirable action (e.g. exercising regularly) even when this action is exposed to internal and external resistance. In this context, volition has an important role for participation in sports such as for participation in exercise activities for recreational and health purposes. The *volition in exercise* is based on the Rubicon model of action phases [22] and on the personality systems interaction theory [24] that have been used as a theoretical framework for understanding volition [25]. It was well documented that to predict exercise behaviour, the measurement instrument should be as domain-specific as possible [26]. Therefore, the questionnaire that assesses volition in the exercise context, that investigates about the power of “willingness to do”, and that is directly focused on the active lifestyle is the Volition in Exercise Questionnaire (VEQ) [26]. This questionnaire was created in English [26] and then was translated and validated into German [27]. At the moment, there is no available instrument for measuring volition in exercise in Italian language; hence there is a need for such an instrument. The aim of this study was therefore to translate and validate the Italian version of the Volition in Exercise Questionnaire (VEQ-I). This will extend the use of the VEQ to a European language other than English and German and will facilitate research into adults’ physical exercise-specific motivation and volition among the Italian population, with the opportunity to compare it with UK and German sample.

In order to validate the questionnaire in Italian Language, after translation, we assessed its construct validity and its internal consistency. Moreover, to examine the relationship between volition and behavior and to better explore the construct validity of the VEQ-I, we assessed its concurrent validity with PSB-SPE and EMI-2.

## Materials and methods

### Participants

Five hundred thirty-four first-year Italian native-language students at the University of Rome “Foro Italico” (391 males, 143 females), aged between 19 and 44 years (21.04 ± 2.27 ys) volunteered to participate in the study. They were recruited during the University Course “Bases of Motor Activities” by the authors, after an oral explanation of the project during the lessons. The institutional review board of the University of Rome “Foro Italico” approved this investigation. All participants have provided their written informed consent before participation in the study.

### Measures

#### Volition in exercise questionnaire

The VEQ was developed by Elsborg et al. [26] to assess volition in physical exercise. The Italian version of the questionnaire is composed by eighteen items scored on a numeric rating ranging from 0 “it doesn’t match at all” to 3 “exactly matches”. The VEQ measures six aspects of volition: four volitional inhibition factors (VI), which hamper an individual’s goal achievement, and two volitional facilitation factors (VF), which facilitate goal attainment. Specifically, the six subscales of the VEQ are: *Reasons* (VI), *Postponing Training* (VI), *Unrelated Thoughts* (VI), *Self-Confidence* (VF), *Approval from Others* (VI), and *Coping with Failure* (VF). *Reasons* (items 6, 16, e.g., *“I think a lot about my reason for participating in my exercise activity”*/*“Penso molto alle ragioni che mi spingono a fare attività fisica”*) refers to the extent of reasons that the individual considers for participating in the exercise activity. *Postponing Training* (items 2, 9, 15, 18, e.g., *“I only begin my exercise activity when I am pressured to it”/“Inizio l’attività fisica solo quando sono costretto”*) refers to the individual’s tendency to procrastinate the exercise activity. *Unrelated Thoughts*, (items 3, 10, 12, e.g., *“During my exercise activity*, *I am disturbed by thoughts that are not related to the activity itself”/“Mentre faccio attività fisica*, *sono disturbato/a da altri pensieri che non riguardano l’attività stessa”*) refers to the extent to which the individual can concentrate by excluding undesirable emotions and thoughts. *Self-Confidence* (items 4, 5, 11, e.g., *“I believe in my own ability to do well in my exercise activity”/“Credo nella mia capacità di poter svolgere bene l’attività fisica”*) refers to the individual’s feeling to succeed in the exercise activity. *Approval from Others* (items 1, 8, 14, e.g., *“I adapt to others during my exercise activity”/“Mi adatto agli altri durante la mia attività fisica”*) refers to what extent the individual is inclined to other people’s opinions in relation to physical exercise. And finally, *Coping with Failure* (items 7, 13, 17, e.g., *“If I make a mistake during my exercise activity*, *I am quick to improve my effort”/“Se commetto un errore durante l’attività fisica*, *mi sforzo immediatamente per migliorare”*) refers to what extent the individual can learn from a failure experience.

The Psychobiosocial States in Physical Education (PBS-SPE) and the Exercise Motivations Inventory (EMI-2) questionnaires were used to assess the concurrent validity of the VEQ-I.

#### Psychobiosocial States in Physical Education (PBS-SPE)

In this paper we used the Italian 14-item list of pleasant/functional (7 items) and unpleasant/dysfunctional descriptors (7 items) to assess psychobiosocial states (PBS) similarly to the PBS questionnaire used with athletes during COVID-19 lockdown [28] and based on the initial 20-item list of pleasant/functional (10 items) and unpleasant/dysfunctional descriptors (10 items) to assess psychobiosocial states in physical education and sport domain [29,30]. Descriptors were derived from a number of adjectives pertaining to each of the seven psychobiosocial components hypothesized within the IZOF model [31] and established on obtainable lists of descriptors used to consider emotional experiences in youth sport and physical education [32,33]. Each item (discrete psychobiosocial state) was composed from two to five descriptors to convey a faultless and forthright representation of an emotional experience associated to the physical education context. Participants were asked to assess each item on a 5-point scale, ranging from 0 “not at all” to 4 “very, very much”, thinking of how they usually feel within their physical education context.

#### Exercise Motivations Inventory—2 (EMI-2)

Markland & Ingledew [34] proposed 51 items to assess motivation states in physical exercise, that are successively assorted as the following 14 subscales: Stress Management, Revitalisation, Enjoyment, Challenge, Social Recognition, Affiliation, Competition, Health Pressures, Health Avoidance, Positive Health, Weight Management, Appearance, Strength and Endurance, and Nimbleness. Participants were asked to indicate whether each statement was true for them personally or would be true for them if they did exercise. Responses were made on a 6-point Likert type scale ranging from 0 “not at all true for me” to 5 “very true for me”.

In this work the second version of the EMI, the EMI-2, has been used. Specifically, the Italian version of the EMI-2 has been used. The EMI-2 was developed to address the problem of non-exercisers and to improve some of the other subscales compared to the first version (EMI). Also this new version involves fourteen subscales. The factorial validity and invariance of the factor structure across gender were thoroughly tested performing confirmatory factor analysis [34]. The EMI-2 has been found to separate between individuals in the stage of change for exercise and to predict change across a longitudinal period in ways that are coherent with self-determination theory [35].

### Procedure

#### Translation procedures

The translation and cultural adaptation of the VEQ-I was conducted using the forward-backward translation method [36] by two Italian English-speaking researchers and a native English speaker with a good command of Italian language. All researchers were knowledge-able about sport and clinical psychology. The original version of the scale was translated independently by the two researchers and then the translated text was discussed extensively. When a consensus on a pre-version of the questionnaire was reached, the questionnaire was reverse translated by the native English speaker. The original scale in English, the translated and back-translated versions were examined carefully for accuracy. Just a few minor discrepancies on the syntax emerged. These were discussed until agreement on the changes was reached. The Italian final version of the VEQ (VEQ-I) is reported in S1 Appendix (it can be printed and administered directly).

We investigated the construct validity and the internal consistency of the VEQ-I, and its concurrent validity with PBS-SPE and EMI-2.

#### Questionnaires administration

Participants were recruited from the first-year University course “Basi delle Attività Motorie” (Basics of Movement Activities course) of the University of Rome “Foro Italico”. They were invited to complete the questionnaires by an online survey platform (Google Forms) before starting the academic lesson. Participation was anonymous, voluntary, and required online consent. Students were introduced to the topic and advised of the anonymous and voluntary nature of the study. Each participating student completed the questionnaires in about 30 minutes.

The questionnaires administered through Google Forms are available under request.

#### Statistical analysis

We investigated the construct validity, the internal consistency, and the concurrent validity of the VEQ-I with EMI-2 and PBS-SPE. To explore the construct validity, or the factor structure of the scales, we conducted the confirmatory factor analysis (CFA) on all the 18 VEQ-I items. The CFA model was evaluated according to different fit indices: standardized root mean square residual (SRMR), root mean square error of approximation (RMSEA), Tucker Lewis fit index (TLI) and comparative fit index (CFI). An acceptable fit was inferred when SRMR value was less than 0.05 [37] and RMSEA value was less than 0.08 [38]. TLI and CFI values greater than 0.90 were considered reflective of good fitting models [39].

The reliability of the VEQ-I was assessed in terms of internal consistency using Cronbach’s alpha (α), that is defined as

$$\alpha =\frac{N}{N-1}(1-\frac{\sum_{i}^{N}\sigma_{Yi}^{2}}{\sigma_{X}^{2}})$$

where X = Y_1_ + Y_2_ + …+ Y_N_ is the quantity we want to measure, N are the components. Usually, a value above 0.7 is satisfactory [40]. Cronbach’s alpha can be interpreted as the expected correlation between the scores obtained by two random samples made up of K items [40]. Omega is an alternative estimated reliability coefficient more appropriate than alpha for psychological research [41]. Alpha and omega, and their Confidence Intervals (CIs), were calculated on each subscale separately.

The connection between volition and behavior has previously been shown in other areas such as applied psychology [25,42]. In this paper, concurrent validity was assessed using a Pearson correlation among the subscales of VEQ-I measures and those of EMI-2. Moreover, as the use of Pearson’s correlation with rating scales is controversial [43], we run also Spearman’ rho and Kendall’s tau coefficients.

Statistical analysis was performed using SPSS software 25.0.

## Results

### Confirmatory factor analysis (CFA)

**Fig 1:**
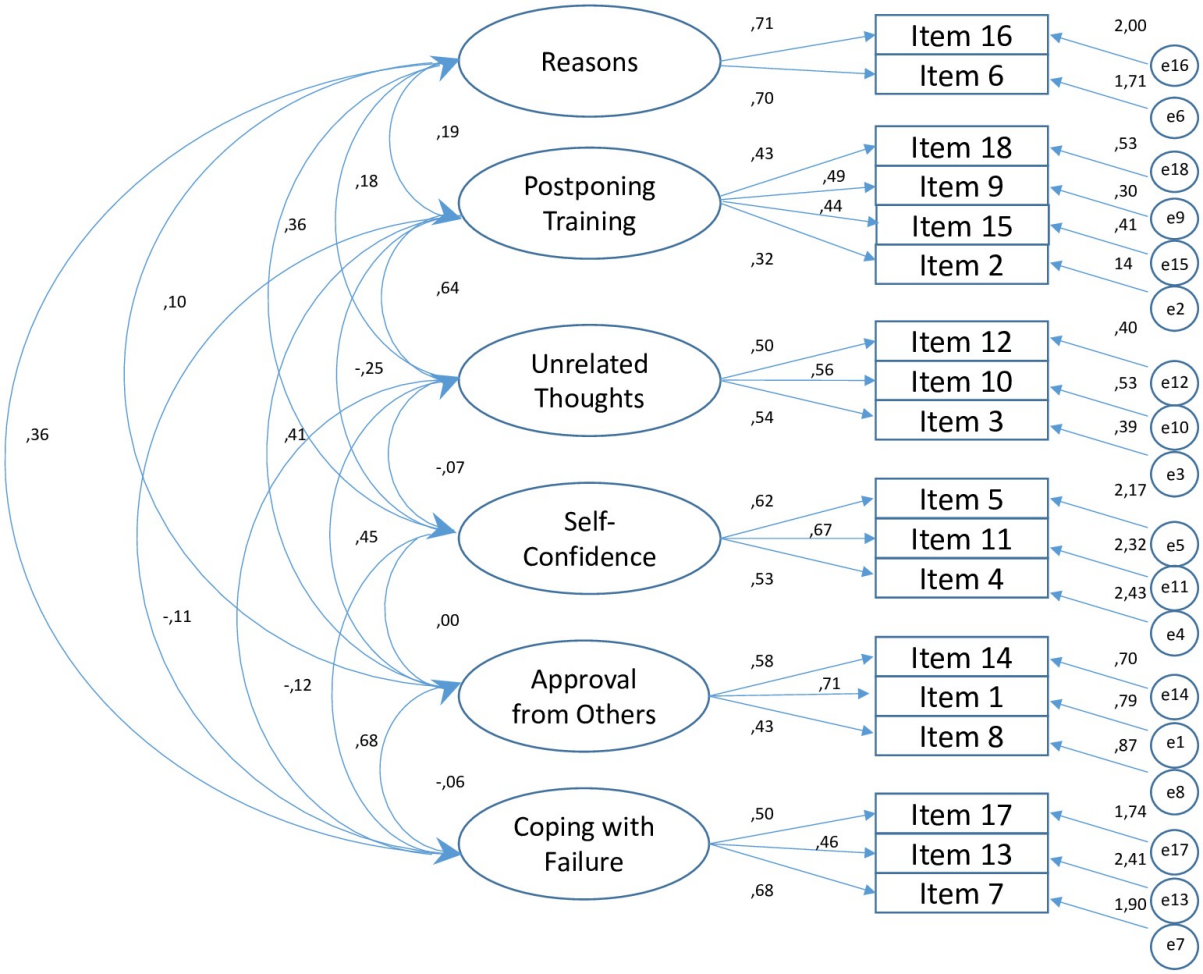
Standardized factor loadings and covariances of the confirmatory factor analysis (CFA).

Construct validity of factor model identified in the Italian version reported acceptable fit indices (CFI = 0.960; TLI = 0.943; RMSEA = 0.039; and SRMR = 0.040). and, consequently, they confirmed the original six-factor structure of the eighteen-item scale also for VEQ-I (Fig 1).

Specifically, the six subscales were: *Reasons* (items 6, 16), *Postponing Training* (items 2, 9, 15, 18), *Unrelated Thoughts* (items 3, 10, 12), *Self-Confidence* (items 4, 5, 11), *Approval from Others* (items 1, 8, 14), *Coping with Failure* (items 7, 13, 17; see S2 Appendix). The main statistical descriptors (mean (M), standard deviation (SD), variance, skewness, and kurtosis) of the items and subscales are reported in Table 1.

**Table 1:** Main statistical descriptors of the items and subscales.

Items and subscales	M	SD	Variance	Skewness	Kurtosis
VI Reasons	1.86	0.81	0.66	-0.320	-0.66
Item 6	1.71	0.92	0.853	-0.194	-0.822
Item 16	2.00	0.89	0.783	-0.502	-0.59
VI Postponing Training	0.35	0.48	0.228	2.074	5.277
Item 2	0.14	0.42	0.179	3.599	15.188
Item 9	0.30	0.65	0.417	2.295	4.925
Item 15	0.41	0.67	0.449	1.639	2.39
Item 18	0.53	0.73	0.533	1.270	1.066
VI Unrelated Thoughts	0.44	0.57	0.328	1.512	2.598
Item 3	0.39	0.65	0.422	1.881	3.85
Item 10	0.53	0.67	0.444	1.178	1.377
Item 12	0.40	0.61	0.366	1.423	1.902
VF Self-Confidence	2.30	0.66	0.436	-0.758	-0.052
Item 4	2.43	0.70	0.488	-0.986	0.306
Item 5	2.17	0.78	0.614	-0.612	-0.272
Item 11	2.32	0.77	0.588	-0.904	0.209
VI Approval from Others	0.79	0.68	0.456	0.775	0.011
Item 1	0.79	0.89	0.785	0.859	-0.201
Item 8	0.87	0.86	0.745	0.658	-0.437
Item 14	0.70	0.82	0.679	0.971	0.189
VF Coping with Failure	2.01	0.59	0.348	-0.634	0.442
Item 7	1.90	0.75	0.561	-0.359	-0.074
Item 13	2.41	0.72	0.511	-0.978	0.274
Item 17	1.74	0.71	0.506	-0.217	-0.087

Note. M = mean, SD = standard deviation.

### Reliability (internal consistency)

Internal consistency of the questionnaire was investigated for each subscale of the VEQ-I using Cronbach’s alpha and omega coefficients. Both Cronbach’s alpha and omega values were acceptable in all the six subscales as reported in Table 2.

**Table 2:** Reliability values of VEQ-I in 6 subscales.

VEQ-I	Items	α (95%CI) [min-max]	ω (95%CI) [min-max]
Reasons (VI)	6–16	0.76 [0.71–0.80]	0.76 [0.71–0.80]
Postponing Training (VI)	2-9-15-18	0.76 [0.70–0.82]	0.76 [0.71–0.80]
Unrelated Thoughts (VI)	3-10-12	0.87 [0.84–0.90]	0.88 [0.84–0.90]
Self-Confidence (VF)	4-5-11	0.85 [0.81–0.87]	0.85 [0.82–0.88]
Approval from Others (VI)	1-8-14	0.70 [0.64–0.74]	0.72 [0.66–0.76]
Coping with Failure (VF)	7-13-17	0.74 [0.69–0.78]	0.74 [0.70–0.78]

Note. VEQ-I = Volition in Exercise Questionnaire—Italian version, α = Cronbach’s alpha values, ω = coefficient omega.

### Criterion (concurrent) validity

To assess concurrent validity, we examined the correlations among the VEQ-I and the two questionnaires EMI-2 and PBS-SPE. The Pearson correlation coefficients were reported in Table 3.

**Table 3:** Pearson’s correlation.

	VEQ-I					
	VF	VI	VI	VI	VI	VF
	Coping with Failure	Approval from Others	Reasons	Unrelated Thoughts	Postponing Training	Self-Confidence
**EMI-2**						
Affiliation		0.165**				
Appearance	0.118**		0.138**			0.098*
Challenge	0.140**		0.173**			0.223**
Competition						0.151**
**Enjoyment**	0.123**		0.106*		-0.109*	0.207**
Health Pressures		0.091*	0.097*			
Ill-health Avoidance	0.103*		0.151**			
Nimbleness	0.104*		0.116**			0.104*
Positive Health	0.149**		0.164**			0.179**
**Revitalization**	0.115**		0.098*		-0.101*	0.109*
Social Recognition	0.129**	0.133**	0.118**			0.188**
Strength & Endurance	0.166**		0.136**			0.163**
Stress Management	0.119**		0.188**			0.161**
Weight Management	0.084*		0.117**			
**PBS-SPE**						
Pleasant/Functional (+)	0.249**		0.099*	-0.149**	-0.155**	0.243**
Motivational (+)	0.199**	-0.093*		-0.168**	-0.181**	0.187**
Volitional (+)	0.124**				-0.115**	0.177**
Unpleasant/Dysfunctional (-)	-0.161**	0.207**		0.199**	0.262**	-0.169**
Motivational (-)	-0.147**	0.131**		0.194**	0.228**	-0.153**
Volition (-)	-0.113**	0.103*			0.129**	-0.141**

** p<0.001;

* p<0.05.

Note. (+) functional psychobiosocial states; (-) dysfunctional psychobiosocial states.

The correlations among the VEQ-I subscales, EMI-2 and PBS-SPE subscales were positive for VF subscales and were negative for VI subscales as expected (but not for Reasons subscale) and ranged in magnitude from weak to moderately high (see Table 3). Unrelated Thoughts (VEQ-I) was the only subscale that did not correlate with any EMI-2 subscales. Comparable results were obtained when running Spearman’s rho and Kendall’s tau coefficients (see supplemental materials S3 Appendix).

## Discussion

The current study aimed to provide an adaptation and validation of the Volition in Exercise Questionnaire [26] in Italian language. Therefore, we investigated the construct validity, the internal consistency, and the concurrent validity of the scale in a sample of Italian adults. This study can expand the use of the instrument in another culture and language beside the available versions in English and German [26,27]. In addition, this study examined the validity of the VEQ-I beyond the initial results provided by Elsborg et al. [26] and Pfeffer et al. [27], comparing the questionnaire with other available instruments on this topic in Italian language.

A confirmatory factor analysis confirmed the six-factor structure of the English version. The analysis showed model fit indices suggesting a good model fit [37]. This indicates that the translation of the questionnaire was successful and that the VEQ-I is a valid tool. These six factors are in line with the theoretical assumptions of the Rubicon model and the personality systems interaction theory and with the results of the other version of the instrument [26,27]. The fit indices of the models were good and factor loadings of the VEQ-I items were comparable for the Italian version with the general model and previous versions.

Furthermore, given that reliability is a crucial element for validity, we calculated internal consistency for each questionnaire subscale using Cronbach’s alpha. Unlike the validation of the German-language version of the questionnaire [27] in which two subscales showed unsatisfactory indices, in our sample reliability was satisfactory for five out of six subscales of the VEQ-I (i.e., Reasons, Postponing Training, Unrelated Thoughts, Self-Confidence and Coping with Failure) and showed acceptable to good Cronbach’s alpha values ranging from 0.738 to 0.874. Approval from others was the only scale to show an internal consistency slightly below the threshold of 0.70 (i.e., 0.695). This might be due to the small number of items included in the scale and it is in line with the results obtained in the development and initial validation of the questionnaire [26]. However, this slightly lower alpha value is not an impediment to using the subscale as it covers important content of the considered construct [44]. Approval from others subscale seems indeed to be important for explaining volitional processes of behaviour enactment [27]. Accordingly, VEQ-I can be considered as overall internally consistent and reliable.

Despite the correlation of Reasons subscale and the lack of correlation of Unrelated Thoughts subscale, we observed an expected correlations trend among VEQ-I, EMI-2 and PBS-SPE subscales. However, despite the generally significant correlations observed, the maximum magnitude value is only moderately high. Therefore, potential overlaps of the volition construct with motivation and psychobiosocial states were no detected. This highlight that the three questionnaires share some common variance but concurrently measure different constructs [42]. We surmise that this finding, especially as concerns the correlations with PBS-SPE subscales, is due to the domain-specific nature of the VEQ-I that is highly related to physical exercise context [45]. Our findings also concur with the theoretical assumption that the realization of a certain health behaviour is a result of motivational as well as volitional mechanisms and that motivation is necessary but could not sufficiently enact a physical exercise behaviour [45]. Hence, as also proposed by Fuchs et al. [46], motivational processes need to be supplemented by volitional processes. This further provides explanatory value to volitional measures in the adherence mechanisms to physical exercise context.

## Conclusions

The purpose of this study was to provide the validation of the Volition in Exercise Questionnaire in Italian language (VEQ-I).

The VEQ-I was comprised of eighteen items that correspond to the six-factor structure of the original version [26] and the German version [27]; the six factors were Reasons, Postponing Training, Unrelated Thoughts, Self-Confidence, Approval from Others, Coping with Failure.

VEQ-I showed a construct validity because it was able to predict physical exercise participation: the eighteen items well distributed in six subscales supported the six-factor structure of the questionnaire.

### Implications

The validation of the VEQ-I is in line with the growing interest on cognitive and emotional responses to physical exercise adherence and with an holistic, multidisciplinary and multidimensional approach intended to improve the adherence to the physical exercise prescription and to obtain satisfactory training results. Validating the Italian version of VEQ can in turn encourage the validation of other questionnaires usually used to evaluate athlete’s psychological readiness. Moreover, the questionnaire could be used for evaluating both subjects with diseases (e.g., obesity) and who have to lead a “treatment without drugs” (e.g., high level athletes).

#### Limitations and future research directions

Additional research is recommended to better examine the psychometric characteristics of the questionnaire and to address some limitations of the current study. We used a quite homogeneous sample of the reference population (i.e., students) where gender was not equally represented, and age was characterized by low SD. Thus, future studies should engage a larger number of participants also examining gender and age-based differences. Moreover, research should be extended to different types of leisure-time physical activity or to the field of clinical physical exercise prescription. In this field, as mentioned previously in this paper, the adherence plays a fundamental role for individuals suffering from metabolic diseases such as obesity.

## Supporting information

S1 Appendix Italian version of the Volition in Exercise Questionnaire (VEQ-I). (DOCX)

S2 Appendix 18 items and 6 sub-scales of VEQ-I. (DOCX)

S3 Appendix Spearman’s and Kendall’s correlation. (DOCX)

## Abbreviations

CFA: confirmatory factor analysis
CFI: comparative fit index
EMI-2: Exercise Motivations Inventory
PBS-SPE: Psychobiosocial States in Physical Education
RMSEA: root mean square error of approximation
SRMR: standardized root mean square residual
TLI: Tucker Lewis fit index
VEQ-I: Italian version of the Volition in Exercise Questionnaire
VF: volitional facilitation factor
VI: volitional inhibition factor
α: Cronbach’s alpha values
ω: coefficient omega
