# Supplementary material for: Initial validation of the Italian version of the Volition in Exercise Questionnaire (VEQ-I)
Source: PLoS One. 2021 Apr 9;16(4):e0249667. doi: 10.1371/journal.pone.0249667 (PMC8034746; doi:10.1371/journal.pone.0249667)
Supplement: S1 Appendix — (DOCX) [file pone.0249667.s001.docx]

**Appendix A – Italian version of the Volition in Exercise Questionnaire (VEQ-I).**

Questionario di volizione nell’esercizio fisico

Assegnare un punteggio alla corrispondenza di ciascuna frase

*(0 = Non corrisponde affatto; 3 = Corrisponde esattamente)*

| 1. Sento di dover soddisfare le aspettative degli altri durante la mia attività fisica. | 0 | 1 | 2 | 3 |
| --- | --- | --- | --- | --- |
| 1. Inizio l’attività fisica solo quando sono costretto. | 0 | 1 | 2 | 3 |
| 1. Mentre faccio attività fisica, spesso trovo difficoltà a concentrarmi perché comincio a pensare ad altre cose. | 0 | 1 | 2 | 3 |
| 1. Credo nella mia capacità di poter svolgere bene l’attività fisica. | 0 | 1 | 2 | 3 |
| 1. Sono convinto di essere in grado di svolgere attività fisiche molto intense. | 0 | 1 | 2 | 3 |
| 1. Durante l’attività fisica mi concentro spesso sulle ragioni che mi spingono a farla. | 0 | 1 | 2 | 3 |
| 1. Quando commetto un errore durante l’attività fisica, riesco a superarlo rapidamente. | 0 | 1 | 2 | 3 |
| 1. Mi adatto agli altri durante la mia attività fisica. | 0 | 1 | 2 | 3 |
| 1. Aspetto di iniziare la mia attività fisica fino a quando non ho più scuse per non farla. | 0 | 1 | 2 | 3 |
| 1. Mentre faccio attività fisica, sono disturbato/a da altri pensieri che non riguardano l’attività stessa. | 0 | 1 | 2 | 3 |
| 1. Credo che la mia volontà sia abbastanza forte da poter svolgere attività fisiche molto intense. | 0 | 1 | 2 | 3 |
| 1. Mentre faccio attività fisica, trovo difficile concentrarmi su quello che sto facendo perché i miei pensieri vanno altrove. | 0 | 1 | 2 | 3 |
| 1. Se commetto un errore durante l’attività fisica, mi sforzo immediatamente per migliorare. | 0 | 1 | 2 | 3 |
| 1. Ho paura di ciò che gli altri pensano di me se non eseguo l’attività fisica così come previsto. | 0 | 1 | 2 | 3 |
| 1. Spesso devo farmi coraggio per praticare attività fisica. | 0 | 1 | 2 | 3 |
| 1. Penso molto alle ragioni che mi spingono a fare attività fisica. | 0 | 1 | 2 | 3 |
| 1. Anche quando la mia attività fisica non sta procedendo al meglio, riesco facilmente a raddrizzare il tiro. | 0 | 1 | 2 | 3 |
| 1. Aspetto fino all’ultimo momento prima di iniziare la mia attività fisica. | 0 | 1 | 2 | 3 |
