# Supplementary material for: Initial validation of the Italian version of the Volition in Exercise Questionnaire (VEQ-I)
Source: PLoS One. 2021 Apr 9;16(4):e0249667. doi: 10.1371/journal.pone.0249667 (PMC8034746; doi:10.1371/journal.pone.0249667)
Supplement: S2 Appendix — (DOCX) [file pone.0249667.s002.docx]

**Appendix B – 18 items and 6 sub-scales of VEQ-I.**

| **Items** | **Latent Factors** |
| --- | --- |
| (16) I think a lot about my reason for participating in my exercise activity | Reasons |
| (6) During my exercise activity I often focus on my reason for participating |  |
| (18) I wait until the last minute before I begin in my exercise activity | Postponing Training |
| (9) I wait to embark on my exercise activity until there is no way around it |  |
| (15) I often experience that I really need to pull myself together to participate in my exercise activity |  |
| (2) I only begin my exercise activity when I am pressured to it |  |
| (12) During my exercise activity, I find it difficult to concentrate because my thoughts drift to other things than the activity itself | Unrelated Thoughts |
| (10) During my exercise activity, I am disturbed by thoughts that are not related to the activity itself |  |
| (3) During my exercise activity, I often find it difficult to concentrate because I start thinking of things not connected to the activity itself |  |
| (5) I am convinced that I am able to carry out strenuous exercise activities | Self-Confidence |
| (11) I believe that my will is strong enough to carry out strenuous exercise activities |  |
| (4) I believe in my own ability to do well in my exercise activity |  |
| (14) I am afraid of what others think of me if I do not perform as expected in my exercise activity | Approval from Others |
| (1) I feel I have to meet others’ expectations during my exercise activity |  |
| (8) I adapt to others during my exercise activity |  |
| (17) Even though my exercise activity is not going well, it is easy for me to get back on track | Coping with Failure |
| (13) If I make a mistake during my exercise activity, I am quick to improve my effort |  |
| (7) When I make a mistake during my exercise activity, I quickly move on |  |

Note. The numbers in brackets indicates the corresponding item in the Italian version of the Volition in Exercise Questionnaire (VEQ-I).
