# Supplementary material for: Initial validation of the Italian version of the Volition in Exercise Questionnaire (VEQ-I)
Source: PLoS One. 2021 Apr 9;16(4):e0249667. doi: 10.1371/journal.pone.0249667 (PMC8034746; doi:10.1371/journal.pone.0249667)
Supplement: S3 Appendix — (DOCX) [file pone.0249667.s003.docx]

**Appendix C.**

**Spearman’s correlation.**

|  | | **VEQ-I** |  |  |  |  |  |
| --- | --- | --- | --- | --- | --- | --- | --- |
|  | | VF | VI | VI | VI | VI | VF |
|  | Coping with Failure | | Approval from Others | Reasons | Unrelated Thoughts | Postponing Training | Self-Confidence |
| **EMI-2** |  | |  |  |  |  |  |
| Affiliation | 0.107* | | 0.184** |  |  |  | 0.084* |
| Appearance | 0.103* | |  | 0.139** |  |  | 0.088* |
| Challenge | 0.170** | |  | 0.182** |  | -0.089* | 0.254** |
| Competition | 0.091* | | 0.111** |  |  |  | 0.178** |
| Enjoyment | 0.126** | |  | 0.128** |  | -0.144** | 0.225** |
| Health Pressures |  | | 0.104* |  | 0.098* | 0.102* |  |
| Ill-health Avoidance | 0.112** | |  | 0.143** |  |  |  |
| Nimbleness | 0.131** | |  | 0.117** |  |  | 0.119** |
| Positive Health | 0.172** | |  | 0.165** |  |  | 0.171** |
| Revitalization | 0.122** | |  | 0.109* |  | -0.108* | 0.104* |
| Social Recognition | 0.146** | | 0.161** | 0.115** | 0.104* |  | 0.211** |
| Strength & Endurance | 0.186** | |  | 0.156** |  |  | 0.176** |
| Stress Management | 0.122** | |  | 0.195** |  |  | 0.178** |
| Weight Management |  | |  | 0.103* |  |  |  |
| **PBS-SPE** |  | |  |  |  |  |  |
| Pleasant/Functional (+) | 0.225** | |  | 0.095* | -0.135** | -0.200** | 0.251** |
| Motivational (+) | 0.172** | |  |  | -0.150** | -0.205** | 0.170** |
| Volitional (+) | 0.121** | |  |  | -0.086* | -0.144** | 0.182** |
| Unpleasant/Dysfunctional (-) | -0.173** | | 0.170** |  | 0.218** | 0.282** | -0.164** |
| Motivational (-) | -0.168** | | 0.096* |  | 0.196** | 0.251** | -0.166** |
| Volition (-) | -0.115** | |  |  |  | 0.117** | -0.155** |

** p<0.001; * p<0.05

**Kendall’s correlation.**

|  | | **VEQ-I** |  |  |  |  |  |
| --- | --- | --- | --- | --- | --- | --- | --- |
|  | | VF | VI | VI | VI | VI | VF |
|  | Coping with Failure | | Approval from Others | Reasons | Unrelated Thoughts | Postponing Training | Self-Confidence |
| **EMI-2** |  | |  |  |  |  |  |
| Affiliation |  | | 0.165** |  |  |  |  |
| Appearance | 0.118** | |  | 0.138** |  |  | 0.098* |
| Challenge | 0.140** | |  | 0.173** |  |  | 0.223** |
| Competition |  | |  |  |  |  | 0.151** |
| Enjoyment | 0.123** | |  | 0.106* |  | -0.109* | 0.207** |
| Health Pressures |  | | 0.091* | 0.097* |  |  |  |
| Ill-health Avoidance | 0.103* | |  | 0.151** |  |  |  |
| Nimbleness | 0.104* | |  | 0.116** |  |  | 0.104* |
| Positive Health | 0.149** | |  | 0.164** |  |  | 0.179** |
| Revitalization | 0.115** | |  | 0.098* |  | -0.101* | 0.109* |
| Social Recognition | 0.129** | | 0.133** | 0.118** |  |  | 0.188** |
| Strength & Endurance | 0.166** | |  | 0.136** |  |  | 0.163** |
| Stress Management | 0.119** | |  | 0.188** |  |  | 0.161** |
| Weight Management | 0.084* | |  | 0.117** |  |  |  |
| **PBS-SPE** |  | |  |  |  |  |  |
| Pleasant/Functional (+) | 0.249** | |  | 0.099* | -0.149** | -0.155** | 0.243** |
| Motivational (+) | 0.199** | |  |  | -0.168** | -0.181** | 0.187** |
| Volitional (+) | 0.124** | |  |  |  | -0.115** | 0.177** |
| Unpleasant/Dysfunctional (-) | -0.161** | | 0.207** | 00.066 | 0.199** | 0.262** | -0.169** |
| Motivational (-) | -0.147** | | 0.131** |  | 0.194** | 0.228** | -0.153** |
| Volition (-) | -0.113** | |  |  |  | 0.129** | -0.141** |

** p<0.001; * p<0.05
